# ATP increases the migration of microglia across the brain endothelial cell monolayer

**DOI:** 10.1042/BSR20160054

**Published:** 2016-04-15

**Authors:** Tomoji Maeda, Manato Inagaki, Yu Fujita, Takehiro Kimoto, Chiaki Tanabe-Fujimura, Kun Zou, Junjun Liu, Shuyu Liu, Hiroto Komano

**Affiliations:** * Department of Neuroscience, School of Pharmacy, Iwate Medical University, 2-1-1 Yahaba-cho, Shiwa-gun, Iwate 020-3994, Japan

**Keywords:** adenosine 5′-triphosphate (ATP), blood–brain barrier, matrix metalloproteinase, microglia

## Abstract

The cerebral microcapillary endothelium, known as the blood–brain barrier (BBB), acts as a barrier between the blood and the interstitial fluid of the brain. The BBB therefore controls the passage of nutrients into the central nervous system (CNS). Microglia show a specific affinity for migration into the CNS, and this migration appears to occur independently of BBB integrity. To study the migration of microglia across the BBB, we developed an *in vitro* co-culture system of mouse brain endothelial cells (MBECs) and Ra2 microglia using Transwell inserts. We first investigated the influence of microglia or ATP, a microglial chemotactic factor, on MBEC barrier integrity. The addition of microglia or ATP led to the disruption of the MBEC monolayer and significantly decreased barrier function as measured by trans-endothelial electrical resistance (TEER) and electric cell–substrate impedance sensing (ECIS). Furthermore, ATP promoted the migration of microglia but not macrophages across the MBEC monolayer. An inhibitor of matrix metalloproteinases (MMPs) decreased the transmigration of microglia in our system, indicating that MMPs play a role in microglial chemotaxis. We specifically identify a role for microglia-derived MMP-2. In conclusion, we offer evidence that microglia migration across the brain endothelial cell monolayer is increased in the presence of ATP in a manner that involves MMP secretion.

## Abstract (precis)

To elucidate the mechanism of microglial migration across the blood–brain barrier (BBB), we developed an *in vitro* co-culture system and analysed real-time BBB integrity during transmigration. We show that ATP promotes microglia transmigration via a mechanism involving microglial matrix metalloproteinases (MMPs).

## INTRODUCTION

The blood–brain barrier (BBB) plays a critical role in the active transport of nutrients into the brain; it also controls the passage of xenobiotics and pathogens in order to maintain the homoeostasis and function of the central nervous system (CNS). However, during CNS inflammation, blood-borne immune cells access the CNS via the BBB [1,2]. The brain microvessel endothelial cells that comprise the BBB are closely interconnected via special cell–cell junctions such as the tight junction [3], which, along with astrocytes, pericytes and neurons, form the neurovascular unit [4].

Microglia are macrophage-like cells that serve multiple functions in the CNS. Microglia play important roles in the development, differentiation and maintenance of neural cells via phagocytic activity and the production of enzymes, cytokines and trophic factors [5]. Although activated microglia exhibit a phenotype similar to that of macrophages in isolated conditions, this activated phenotype differs from macrophages in physiologically relevant *in vivo* and *in vitro* conditions [6,7]. It is known that intra-arterially injected microglia migrate specifically into the brain; however, the mechanism of microglia migration across the brain microvascular endothelium has not been investigated to date [8].

Several factors regulate cell migration. For example, extracellular ATP promotes cell migration by both autocrine and paracrine mechanisms. The release of ATP from apoptotic cells serves as a paracrine ‘find-me’ signal that promotes phagocytic clearance [9]. Previous studies have demonstrated that extracellular ATP promotes the formation of microglial processes that are characteristic of a surveillance state and chemotactic response [10]. Matrix metalloproteinases (MMPs) are a family of zinc-dependent extracellular matrix enzymes that degrade protein and serve as migratory factors: MMP-2 and MMP-9 increase BBB permeability and are produced by microglia and brain microvascular endothelial cells [11].

To explore the mechanism of microglia migration across the brain endothelium, we developed an *in vitro* Transwell co-culture system of mouse brain endothelial cells (MBECs) and mouse microglia (Ra2 cells) or, for comparison, macrophages (RAW264.7 cells). Measurements of trans-endothelial electrical resistance (TEER) enabled us to study the disruption of the MBEC barrier function by microglia. In addition, a new bioanalytical technique, electric cell–substrate impedance sensing (ECIS) [12], allowed us to monitor real-time changes in barrier function and ultimately provide an innovative *in vitro* assay of BBB-like function.

## MATERIALS AND METHODS

### Cell culture

The Ra2 murine microglia cell line (licensed by the Medical and Biological Laboratories, Patent IDUS6.673,6,5; JP3410738; EP10/602,234) was provided to the researchers by Dr Sawada at Nagoya University. Ra2 cells were maintained in Eagle's minimal essential medium (MEM) supplemented with 10% FBS, 5 mg/ml bovine insulin, 0.2% glucose and 1 ng/ml recombinant mouse granulocyte macrophage colony-stimulating factor (GM-CSF; Genzyme) [8]. MBECs [13] and the RAW264.7 murine macrophage cell line were purchased from A.T.C.C. and maintained in Dulbecco's modified Eagle's medium (DMEM) supplemented with 10% FBS, 100 units/ml penicillin and 100 mg/ml streptomycin.

### Construction and generation of the stable MBEC cell line expressing tomato red fluorescent protein

To establish a stable cell line expressing tomato red fluorescent protein, subconfluent MBEC cells were transfected with ptdTomato-N1 construct (Clontech Laboratories) by using Lipofectamine® 2000 Transfection Reagent (Life Technologies) according to the manufacturer's instructions. At 48 h after transfection, the cells were subjected to geneticin selection (at a final concentration of 1000 μg/ml) (Wako) for 1 week. In addition, transfectants were cultured at 1 cell/well in a 96-well dish for 2 weeks, and then five stable cell lines were selected.

### Trans-endothelial electrical resistance measurements

TEER was measured using an epithelial volt–ohm meter (EVOM) equipped with electrodes (World Precision Instruments). The MBEC monolayer TEER was measured before and at 24, 48 and 72 h after the addition of the Ra2 cells. TEER values are presented as the mean±S.E.M.

### Microglia trans-endothelial migration assay

Microglia migration across the MBEC monolayer was evaluated *in vitro* using Transwell inserts with a pore size sufficient to permit cell migration. MBECs, stably expressing tomato red fluorescent protein, were seeded at 2×10^4^ cells/well on the upper side of a Transwell insert (pore size: 3.0 μm; membrane surface area: 0.33 cm^2^; Corning). Seeded Transwell inserts were then placed into 24-well plates. The volumes of the culture medium were 0.2 and 0.9 ml in the upper and lower chambers respectively. The MBEC cultures were maintained for 3 days. A pMX-GFP construct was generated [14] and Ra2 cells were infected with this construct using a retrovirus as previously described [15]. Ra2 cells, stably expressing GFP, were suspended in DMEM supplemented with 10% FBS and added to the upper chamber of Transwell inserts containing an MBEC monolayer (preculture density: 1×10^5^ cells/well). The medium in the lower chamber was replaced with fresh DMEM supplemented with 10% FBS and 100 μM nucleotides. Cultures were incubated at 37°C in a humidified 5% CO_2_ atmosphere for 72 h. A non-specific MMP inhibitor, GM6001, or its inactive control peptide (EMD Millipore) was then applied at a concentration of 100 μM in the upper chamber. During the migration assay, Ra2 cells were observed with an inverted fluorescence microscope (Olympus). Experiments were performed, in triplicate at minimum, using two separate migration chambers, and data are presented as the mean±S.E.M.

### Electric cell–substrate impedance sensing

To evaluate real-time changes in cell morphology including cell–substrate interactions, cell motility and cell layer barrier function, ECIS measurements were taken from MBECs grown on gold-film electrodes as previously described [12]. From continuously recorded impedance spectra, we extracted two ECIS parameters: Rb, representing the electrical resistance of cell–cell contacts, and the impedance of the cell–substrate adhesion zone, represented by the model parameter *α*.

### ELISA

After 48 h of incubation, conditioned media were collected from Ra2 microglia or MBECs and the expression of MMP-2 was measured using a Total MMP-2 Quantikine ELISA Kit (sensitivity: 0.082 ng/ml) according to the manufacturer's specifications (R&D Systems).

### Statistical analysis

Data are presented as means±S.E.M. Statistical analyses were performed using a two-tailed Student's *t* test. Statistical significance was set at *P*<0.05.

## RESULTS

### ATP increases Ra2 migration across the MBEC monolayer

**Figure 1:**
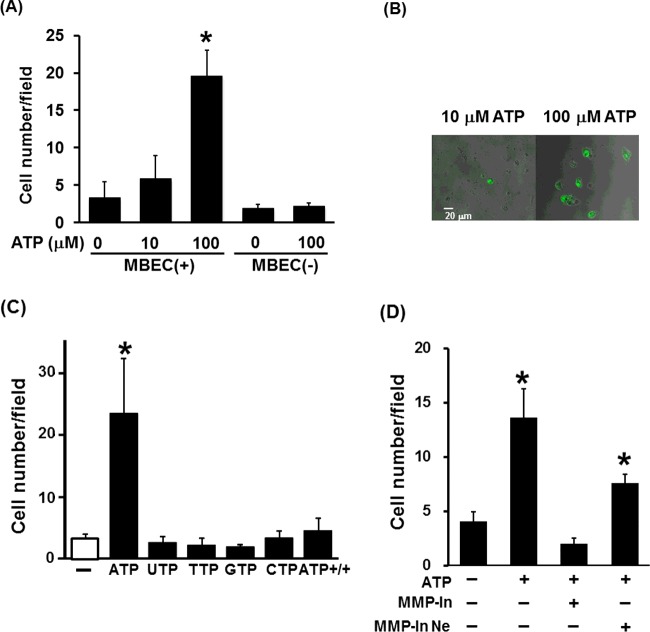
ATP enhances Ra2 cell migration in an MMP activity-dependent manner (**A**) The number of Ra2 cells migrating across an MBEC monolayer per field in the presence (10 μM and 100 μM) or absence (0 μM) of ATP, with (+) or without (-) MBECs. (**B**) Representative images of Ra2 treated with ATP in the Transwell assay. (**C**) The number of Ra2 cells migrating across an MBEC monolayer in the presence of nucleotides. ATP+/+ indicates the presence of ATP in both the upper and lower chambers. (**D**) The number of Ra2 cells migrating across an MBEC monolayer in the presence of ATP (100 μM) and the MMP inhibitor GM6001 (100 μM) or control peptide (100 μM). MMP-In, MMP inhibitor (GM6001); MMP-In Ne, MMP inhibitor negative control (control peptide). Results are presented as the mean±S.E.M. of ten different fields from four independent experiments; *, *P*<0.05 (two-tailed Student's *t* test).

In order to investigate the mechanism of microglia migration across the BBB, we cultured Ra2 microglia and MBECs in a double-chamber Transwell system. We first sought to define the role of ATP in Ra2 migration across the MBEC monolayer. The number of Ra2 cells migrating across the MBEC monolayer significantly increased in the presence of 100 μM ATP compared with non-treated control cells (Figure 1A). Representative images of the transmigration analysis are shown in Figure 1(B). Interestingly, ATP did not increase microglia migration across the Transwell membrane in the absence of co-cultured MBECs (Figure 1A). Other nucleotides had no effect on microglia transmigration (Figure 1C). Moreover, when ATP was added to both chambers in the presence of MBEC, Ra2 microglia did not migrate across the MBEC monolayer in the presence of ATP (Figure 1C). Conversely, macrophages (RAW264.7 cells) did not migrate across the MBEC monolayer in the presence or absence of ATP (results not shown).

### ATP increases the migration of Ra2 across the MBEC monolayer in an MMP activity-dependent manner

To determine whether secreted MMPs facilitate the trans-endothelial migration of microglia, we analysed the effect of an MMP inhibitor (GM6001) or its inactive control peptide on the migration of microglia across the MBEC monolayer in the presence of ATP. The application of GM6001 substantially reduced ATP-induced migration of microglia across the MBEC monolayer (Figure 1D). These data suggest that ATP-induced migration of Ra2 microglia across the MBEC monolayer is MMP-dependent. As it has been reported that GM6001 inhibits MMP-1a, 1b, 2, 3, 8 and 9 [16], we investigated the expression of the corresponding genes in Ra2 microglia and MBECs using a DNA microarray (Supplementary Table S1). The gene expression of MMP-2 was higher than that of other MMPs. To evaluate MMP-2 secretion by Ra2 microglia, culture supernatants were harvested after 48 h and analysed using an ELISA. MMP-2 in Ra2 supernatant was detected at a concentration of 156.3 ± 11.2 ng/mg protein, whereas the level of MMP-2 in MBEC supernatant was approximately 0.28 ± 0.11 ng/mg protein.

### Ra2 migrate paracellularly across the MBEC monolayer

**Figure 2:**
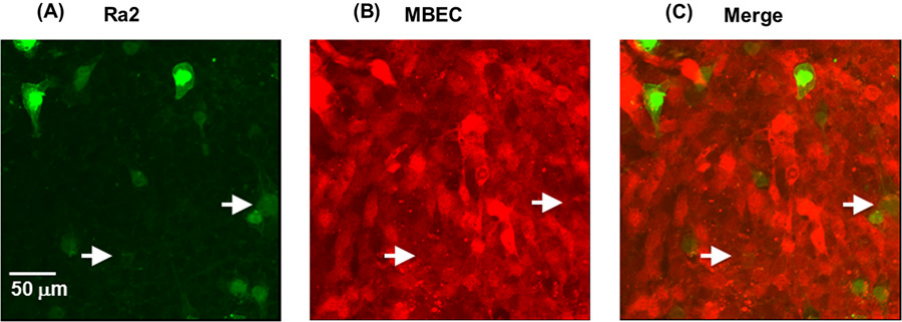
Localization of Ra2 cells in the MBEC monolayer Confocal fluorescence microphotographs of Ra2 cells (green) and MBECs (red) 24 h after the addition of Ra2 cells to MBECs cultures. (**A**–**C**) Representative orthogonal images at two different *z*-positions: focus on Ra2 cells (**A**), focus on MBECs (**B**), (**C**) merge of (A) and (B). Arrows indicate Ra2 cells that were identified under MBECs.

The localization of Ra2 microglia co-cultured with MBECs after 24 h was investigated by confocal fluorescence microscopy. Ra2 microglia were identified primarily in the gaps between MBECs (Figure 2C), and to a lesser extent, under MBECs (Figure 2C, arrows). A detailed analysis of vertically and horizontally cross-sectioned samples revealed that the majority of microglia were attached to the bottom of the dish (Supplementary Figure S1). These results suggest that Ra2 cells migrate paracellularly across the MBEC monolayer.

### MBEC barrier integrity is weakened by ATP treatment and Ra2 co-culture

**Figure 3:**
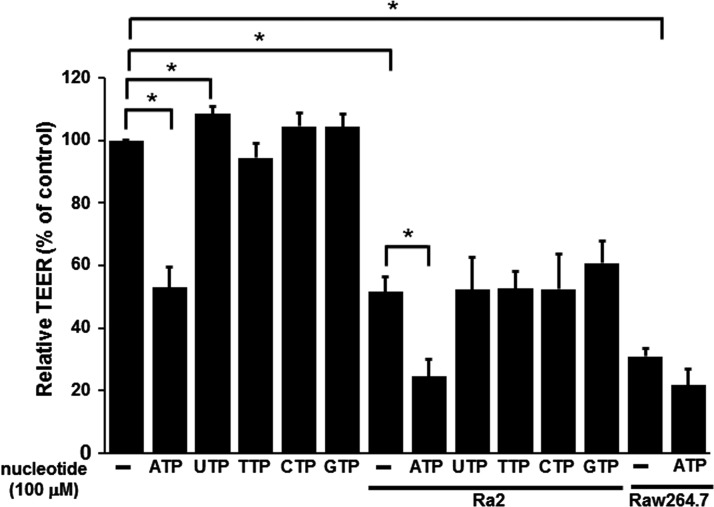
TEER measurements of the MBEC monolayer 48 h after the addition of Ra2 or RAW264.7 cells The integrity of the MBEC monolayer before and after the addition of Ra2 cells or RAW264.7 cells in the presence or absence of nucleotides, as indicated by TEER. Data are expressed as the ratio of the experimental TEER value to the baseline TEER value (MBECs only) and presented as the mean±S.E.M. for three experiments; *, *P*<0.05 (two-tailed Student's *t* test).

**Figure 4:**
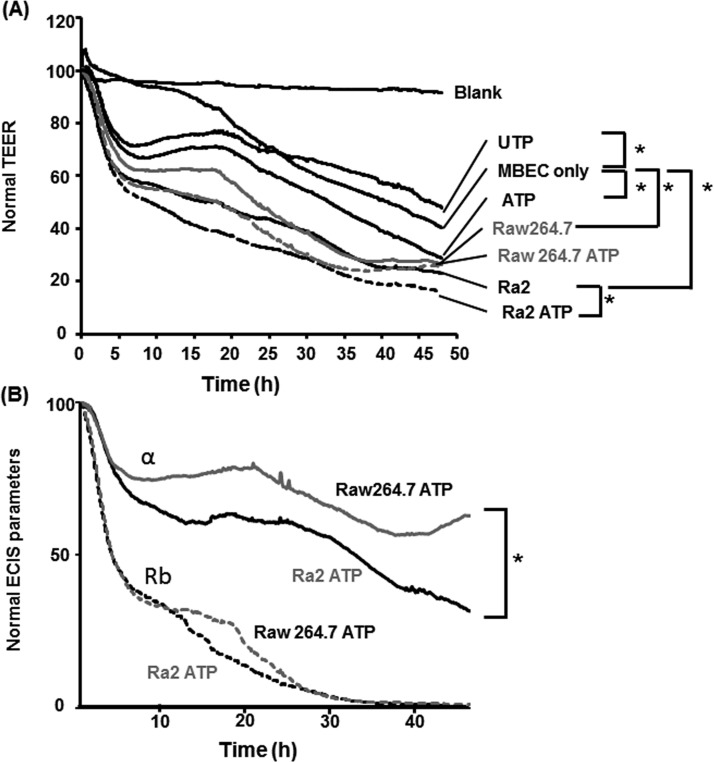
Effect of ATP and Ra2 co-culture on the barrier function of MBECs (**A**) TEER measurements of medium only (blank), an MBEC monolayer grown on gold-film electrodes (control) and MBECs cultured with 100 μM ATP (ATP), 100 μM UTP (UTP), Ra2 cells with 100 μM ATP (Ra2 ATP) or RAW264.7 cells with 100 μM ATP (RAW264.7 ATP) (0 min). (**B**) Effect of Ra2 or RAW264.7 cell co-culture in the presence of ATP on the barrier function of the MBEC monolayer as evaluated by cell–cell contact resistance (Rb) and cell–matrix adhesion impedance (*α*). Normalized TEER (Normal TEER) values and normal ECIS parameters are presented as the mean±S.E.M. for three or four experiments; *, *P*<0.05 (two-tailed Student's *t* test).

TEER was measured to evaluate the influence of ATP treatment and Ra2 or RAW264.7 co-culture on MBEC barrier integrity (Figure 3). TEER values were significantly decreased with ATP treatment, whereas TEER values were significantly increased with UTP treatment. In addition, TEER values were significantly decreased by Ra2 co-culture in the upper chamber of Transwell inserts. These results suggest that the integrity of the MBEC barrier is reduced by Ra2 co-culture and ATP treatment. Although macrophages (RAW264.7 cells) did not cross the MBEC monolayer in the presence or absence of ATP, TEER values were significantly decreased when RAW264.7 cells were co-cultured in the upper chamber of Transwell inserts in the presence or absence of ATP (Figure 3). These results suggest that MBEC barrier integrity is also reduced by RAW264.7 co-culture. To evaluate the different migratory profiles of Ra2 and RAW264.7 cells, we further assessed the effect of ATP treatment on cell–cell contacts and cell–substrate adhesion by means of ECIS. Representative time courses of normal resistance, resistance Rb (cell–cell contacts) and *α* (cell-to-substrate adhesion) of MBECs after co-culture with Ra2 or RAW264.7 cells and ATP treatment are shown in Figure 4. Normal resistances of MBECs treated with ATP or UTP decreased to 71.4±3.0% or 74.3±6.9% within 5 h of application and to 28.7±1.8% or 47.9±2.6% after 48 h respectively (Figure 4A). Consistent with TEER measurements (Figure 3), UTP treatment enhanced the normal resistance of MBECs compared with untreated MBECs after 48 h (47.9±2.6% compared with 40.2±0.7%). Normal resistances of MBECs co-cultured with Ra2 or RAW264.7 cells decreased to 61.2±3.9% or 66.7±4.9% within 5 h and to 22.9±1.4% or 26.2±4.9% after 48 h respectively (Figure 4A). In addition, normal resistances of MBECs co-cultured with Ra2 or RAW264.7 cells and treated with ATP decreased to 57.6±2.8% or 60.7±3.8% within 5 h and to 16.1±1.0% or 25.5±1.9% after 48 h respectively. Normal resistances of MBECs co-cultured with Ra2 cells decreased to 55.7±4.5% after 15 h, and in the additional presence of ATP treatment, normal resistances significantly decreased to 41.2±2.3% after 15 h in comparison with MBEC only, and to 22.9±1.4% or 16.1±1.0% after 48 h respectively. The addition of Ra2 or RAW264.7 cells with ATP decreased the Rb (cell–cell contacts) of MBECs to 46.1±4.8% or 43.4±3.9% within 5 h respectively, and by 100% after 48 h. The *α* (cell-to-substrate adhesion) of MBECs co-cultured with Ra2 and ATP decreased to 76.8±4.5% within 5 h and to 35.6±8.3% after 48 h, whereas the co-culture of MBECs with RAW264.7 cells and ATP decreased *α* to 82.5±8.8% within 5 h and to 62.8±4.8% after 48 h (Figure 4B).

## DISCUSSION

In the present study, we designed an *in vitro* culture system that allows the study of cell migration across an MBEC monolayer on a Transwell membrane, and used this novel assay to define a role for ATP and MMPs in microglia migration across endothelial barriers (e.g. the BBB).

ATP can induce microglial chemotaxis [10]; this effect may underlie the accumulation of microglia in damaged brain regions. Therefore, we tested the effect of ATP on microglia migration across the MBEC monolayer. ATP induced the transmigration of Ra2 microglia in a concentration-dependent manner. Other nucleotides did not affect the transmigration of Ra2 cells. These results suggest that ATP plays a role in Ra2 chemotaxis and migration across the MBEC barrier. Furthermore, ATP decreased the TEER in MBECs, whereas UTP increased the TEER but did not affect transmigration. It has been reported that ATP increases MMP-9 activity [17], and it is therefore possible that ATP induced MMP-9 secretion and the subsequent protease disruption of MBEC junctions, which decreased the TEER. UTP has been reported to act as both a chemotactic agent and a mitogenic factor in vascular endothelial cells [18]. Accordingly, it is possible that UTP stimulated the proliferation of MBECs and produced an increase in barrier function, which increased the TEER.

MMPs play a critical role in multiple CNS processes that range from tissue remodelling during development to the phases of neuroinflammation. The major function of MMPs is the degradation and remodelling of all components of the extracellular matrix. MMPs are effectors of BBB disruption [11], and extensive studies in multiple sclerosis and experimental autoimmune encephalomyelitis have highlighted the activities of MMP-2 [19] and MMP-9 [20] in BBB disruption. Following bilateral common carotid artery occlusion, a disease state characterized by cerebrovascular pathology, MMP-2 activity and, to a lesser extent, MMP-9 activity, is increased in the corpus callosum [21]. Moreover, ATP has been reported to induce microglial chemotaxis via the activation of MMP-9 [22]. Therefore, we hypothesized that MMP-2 secretion underlay the mechanism of Ra2 transmigration. Indeed, the MMP inhibitor GM6001 decreased Ra2 microglia transmigration. Expression of MMP-2 but not MMP-9 was observed in Ra2 microglia using zymography (results not shown) and we observed similar results for gene expression using a DNA microarray. These results suggest that MMP-2 may play a role in disrupting MBEC tight junctions to facilitate Ra2 transmigration. Importantly, ATP treatment did not affect MMP-2 secretion and expression, whereas ATP induced the gene expression of MMP-3 and lipocalin 2/neutrophil gelatinase-associated lipocalin, which are involved in the stability of MMP-9 [23] in MBECs measured via DNA microarray (results not shown). Although there are several non-specific MMP inhibitors that block MMP-2 (e.g. GM6001 and APR100), there are no specific inhibitors for MMP-2. Therefore, it is possible that we overlooked the activity of MMP-9 and other MMPs potentially involved in Ra2 migration across MBECs. In addition, the present study showed that ATP induced the gene expression of serum amyloid A, which has been shown to chemoattract leucocyte and dendritic cells [24], and of bone marrow stromal cell antigen 1 (also known as CD157), which mediates the control of cell migration and diapedesis [25], by using DNA microarray. Therefore, potential chemoattractants derived from ATP-treated MBECs may be involved in Ra2 transmigration.

It has been reported that microglia migrate across the BBB by a transcellular route *in vivo* [26]. However, in the present study, a decrease in the TEER of MBECs resultant from Ra2 co-culture suggests the disruption of the MBEC monolayer and therefore the availability of a paracellular route. In addition, migrating Ra2 cells were identified in the gaps between MBECs. These data suggest that microglia can migrate through the MBEC monolayer by a paracellular route. However, extracellular ATP was present in higher concentrations in our microglia trans-endothelial migration assay, and it has been reported that extracellular nucleotides at higher concentrations are considered pro-inflammatory [9]. Microglia are associated with inflammation. Therefore, this assay system might be an inflammation rather than a normal condition, and two routes may exist in normal conditions.

Although both microglia and macrophages were able to disrupt the MBEC monolayer *in vitro*, only microglia migrated across the MBEC layer in our transmigration assay. Additionally, although the TEER of MBECs decreased similarly following Ra2 or RAW264.7 co-culture, ATP treatment of Ra2 co-cultures had a greater impact on the TEER of MBECs. These data suggest that ATP induces the transmigratory/barrier disruptive activity of Ra2 but not RAW264.7 cells (Figure 3). Finally, observed values for Rb (cell–cell contacts) were the same for Ra2 and RAW264.7 co-culture conditions, whereas the observed *α* values (cell-to-substrate adhesion) were statistically different. Taken together, these results suggest that microglia are capable of penetrating the subendothelial space and migrating across the MBEC monolayer.

It was recently reported that microglia have a radically different expression pattern of purine/pyrimidine receptors from that of peripheral macrophages. Compared with macrophages, microglia express significantly higher levels of purine/pyrimidine receptors that include *P2rx7, P2ry12, P2ry13 and P2ry6*. In contrast, macrophages express significantly higher levels of *P2rx4* [27]. Therefore, the different expression pattern of purine/pyrimidine receptors between microglia and macrophage may be involved in migration across the MBEC monolayer.

In conclusion, we report the establishment of an *in vitro* culture system that mimics the barrier function of the BBB via an MBEC monolayer on Transwell inserts, and demonstrate that ATP promotes MBEC barrier disruption and paracellular transmigration of Ra2 microglia. In addition, Ra2 transmigration occurred in an MMP-dependent fashion. Understanding the mechanism of Ra2 migration across MBECs informs the future design of brain-specific drug delivery systems. Transmigration factors identified in the Ra2-MBEC co-culture system can be expressed in macrophages or dendritic cells; these cells are good candidate vehicles for CNS-targeted gene therapy or drug delivery in future studies.

## Abbreviations

BBB: blood–brain barrier
CNS: central nervous system
DMEM: Dulbecco's modified Eagle's medium
ECIS: electric cell–substrate impedance sensing
MBEC: mouse brain endothelial cell
MMP: matrix metalloproteinase
TEER: trans-endothelial electrical resistance
